# Delayed diagnosis of foreign body on the tongue: case report

**DOI:** 10.5935/1808-8694.20120047

**Published:** 2015-10-20

**Authors:** Sultan Şevik Eliçora, Mehmet Güven

**Affiliations:** a MD (Department of ORL, Sakarya Research and Training Hospital, Sakarya, Turkey); ^2^ Associate Professor (Department of ORL, Sakarya Research and Training Hospital, Sakarya, Turkey)

**Keywords:** dental materials, foreign bodies, tongue

## INTRODUCTION

Upper-airway and digestive system foreign body is frequently seen on otorhinolaryn-gological (ear-nose and throat) region. Deeply located foreign bodies found on the tongue especially on removable part of it are seen quite rare and they generally mimic the malignancy^1^. The foreign bodies such as fishbone, projectile, piece of snaggletooth, piece of nut, piece of pipe, and piece of metallic umbel, seen on the tongue and removed surgically, were reported before^1^, ^2^, ^3^. Early intervention is required because sharp and pungent ones of these foreign bodies cause perforation by migration. This paper aims to present a case of determining a piece of deeply located dental needle with the complaint of a tongue with movable swelling and pain and of its being removed surgically.

## CASE PRESENTATION

72-year-old female patient came to our clinic with the complaint of a tongue with a swelling and pain that existing for a month and moving from time to time. It was learnt that the pain started with a feeling of bitter one month ago while eating rice and it continued increasingly. There is no trauma or operation on head and neck region, dysphagia, or neuromuscular disorder in her medical history. In the patient's physical examination, a mass- lesion which is 1 × 0.2 cm dimensional, well-circumscribed and raised from the surface was observed on the dorsal and right lateral ventral sides of the tongue. It was painful on palpation, well-circumscribed and had a firm consistency. Tongue mucosa was observed regular and on natural color. Other otorhinolaryngology and systemic interventions are normal.

**Figure 1:**
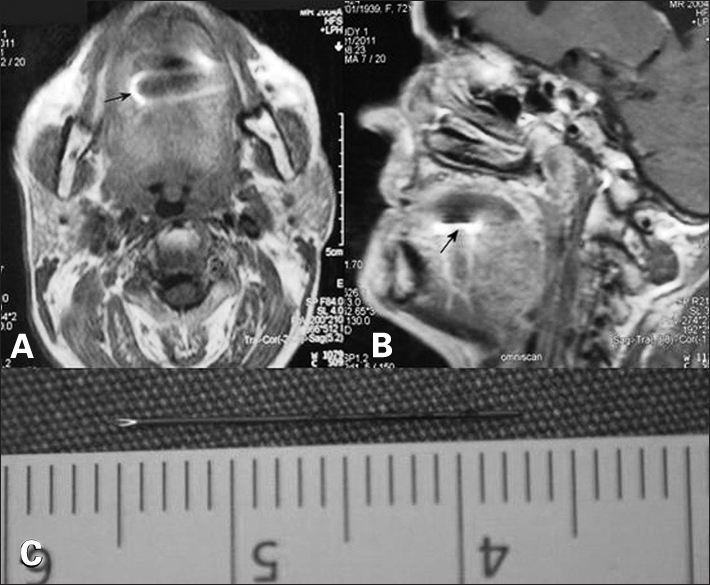
Image of the foreign body on axial (A) and sagittal (B) sections of MR imaging (black arrow). Image of the dental needle that was removed (C).

In the patient's examination of Magnetic Resonance (MR) imaging, a metallic foreign body, which is 1 × 0, 3 cm dimensional linear localized on the 1/3 front part of the lingual muscles and causing condense artifact was observed (Figure 1A-B).

The metallic body was removed surgically by doing nearly 1-cm-incision over the mass on the right lateral of the tongue under local anesthesia. It was examined that the removed foreign body is 18-mm-long piece of dental needle (Figure 1C). The patient was questioned for her past experiences and it was learnt that she had a dental operation 6 months ago.

Neuromuscular or neurosensorial deficit were not observed after 10-month-observation of the patient who recovered from her complaints and did not develop any complication after the operation.

## DISCUSSION

Foreign bodies on the movable part of the tongue are generally superficial and they can be removed easily by the general practitioner or the patients themselves^1^. Yet, deeply located and invisible foreign bodies mimics the malignancy by occurring of the swelling, neuralgic pain, ecchymosis, sub-mental swelling. The patient presented our clinic similarly with the symptoms of swelling and pain on her tongue. Her old age made us to think about malignancy while well-circumscribed swelling and tongue mucosa that is intact and colored with a normal mucosa color set us think about benign pathology.

There may not be found any information about the foreign body on the swelled tongue in the medical history of the patient. A trauma or operation history at the first examination may be missed or forgotten by the patient while questioning the patient^4^. In this case, she presented not a trauma or operation history at the first examination but a sharp ache on her tongue while eating chicken rice approximately one month ago. Understanding that the foreign body was a dental needle, the patient was questioned about her past operational history and she reported that she had a dental operation six months ago but she did not have any problem later on.

The diagnosis of the deeply located and invisible foreign bodies on the tongue is frequently radiological. Most of the foreign bodies are radiopaque but the foreign bodies that are not radiopaque such as wooden, plastics, glasses, fishbone and chicken bones may not be seen as radiographic. It can be useful for the ultrasound diagnosis on this type of foreign bodies^5^. MRI is a valuable method for diagnosis. It is extremely prosperous especially at the discrimination of new structures with inflammatory lesions. Computed tomography and ultrasonography can be used in the cases, in which the percentage of findings foreign body is higher, to identify the shape and size of the pieces^6^.

Even if the foreign bodies on the upper digestive tract are seen easily, few of them are not seen due to their being deeply located and the migration observed on especially sharp metallic foreign bodies might cause serious or even fatal complications^1^.

## CLOSING REMARKS

Consequently, foreign body should be kept in mind for differential diagnosis of the mass lesion, it should be especially questioned in the patient's past history and surgical intervention for the foreign body should be performed as soon as possible.
